# Paediatric Fabry disease: prognostic significance of ocular changes for disease severity

**DOI:** 10.1186/s12886-016-0374-2

**Published:** 2016-11-16

**Authors:** Gisela Kalkum, Susanne Pitz, Nesrin Karabul, Michael Beck, Guillem Pintos-Morell, Rossella Parini, Marianne Rohrbach, Svetlana Bizjajeva, Uma Ramaswami

**Affiliations:** 1 Department of Paediatrics, Helios-Dr-Horst-Schmidt-Kliniken HSK, Ludwig-Erhard-Strasse 100, 65199 Wiesbaden, Germany; 2 Department of Ophthalmology, University Medical Centre, Johannes Gutenberg University, Mainz, Germany; 3 Department of Neuropaediatrics and Inborn Metabolic Disorders (Metabolicum Ruhr), University Children’s Hospital, Centre for Rare Diseases, Ruhr University Bochum, Bochum, Germany; 4 Institute of Human Genetics, University Medical Centre, Johannes Gutenberg University, Mainz, Germany; 5 Department of Paediatrics, Germans Trias i Pujol University Hospital, and Research Institute IGTP, Badalona, Universitat Autònoma de Barcelona, Barcelona, Spain; 6 Rare Metabolic Diseases Unit, Fondazione MBBM, San Gerardo Hospital, Monza, Italy; 7 Division of Metabolism, University Children’s Hospital, Children’s Research Centre, Zurich, Switzerland; 8 Shire, Zug, Switzerland; 9 Lysosomal Disorders Unit, Royal Free London Hospitals NHS Foundation Trust, London, UK

**Keywords:** Agalsidase alfa, Children, Fabry disease, Fabry Outcome Survey, Ocular signs

## Abstract

**Background:**

Ocular signs of Fabry disease can be seen in the first decade of life.

**Methods:**

We examined the occurrence of ocular signs in 232 paediatric patients in the Fabry Outcome Survey (FOS) international registry and looked for relationships between the presence of eye findings and disease severity as measured by the FOS Mainz severity score index (FOS-MSSI).

**Results:**

At least one ocular sign was found in 55/101 (54.5%) girls and 62/131 (47.3%) boys: cornea verticillata in 53/101 (52.5%) girls and 55/131 (42.0%) boys, vessel tortuosity in 17/98 (17.3%) girls and 32/131 (24.4%) boys, and posterior spoke-like lens opacities in 3/97 (3.1%) girls and 2/130 (1.5%) boys. Summary statistics showed higher median (range) age-adjusted FOS-MSSI total score indicating more severe disease in children with eye findings versus those without eye findings (0.5 [−11.0, 20.7] versus −2.3 [−11.1, 18.8]). At least one eye finding was observed in 59.1% of treated and 37.9% of untreated children.

**Conclusions:**

We conclude that the presence of ocular signs, particularly cornea verticillata, correlates with more severe disease as indicated by FOS-MSSI scores in paediatric patients with Fabry disease. Ocular signs appear in roughly half of school-aged children with Fabry disease and are well-recognised as a valuable tool for diagnosis of Fabry disease in children; they also may help identify patients who are at risk for developing early severe manifestations of Fabry disease and who should be further evaluated and closely followed up.

**Electronic supplementary material:**

The online version of this article (doi:10.1186/s12886-016-0374-2) contains supplementary material, which is available to authorized users.

## Background

Fabry disease (OMIM number 301500) is a genetic disorder that typically causes reduced life expectancy in males and even in heterozygous females [1–4]. Functional insufficiency of the lysosomal enzyme α-galactosidase A (Enzyme Commission number 3.2.1.22) leads to progressive accumulation of glycosphingolipids in many tissues, with associated morbidity and mortality [3]. Early diagnosis is important to monitor disease progression; further, timely initiation of enzyme replacement therapy (ERT) before irreversible end organ damage occurs, along with other interventions to treat cardiac and renal dysfunction, may improve patient outcomes [5].

Fabry disease can manifest in childhood [6–9]. As in adults [10, 11], these include acute and chronic neuropathic pain, hypohidrosis, angiokeratoma, gastrointestinal symptoms, and reduced quality of life [6–8, 12, 13]. Fabry disease is also associated with ocular signs, which typically do not impair vision and have been reported to occur as early as the first decade of life [14, 15]; corneal findings have even been described in a 22-week foetus [16]. Characteristic ocular signs of Fabry disease fall into three categories: corneal changes, vascular changes, and posterior spoke-like lens opacities [15, 17, 18]. The cornea may exhibit linear deposits called cornea verticillata (CV) and/or a diffuse corneal haze. Vascular changes in Fabry disease include increased tortuosity of conjunctival and/or retinal vessels, as well as aneurysms of conjunctival vessels. Further, a recent report of 38 individuals with Fabry disease described the presence of vascular tortuosity in 36 (94.7%) and microaneurysms in 10 (26.3%) patients on the external surface of the superior eyelid [19]. Conjunctival aneurysms are associated with glycosphingolipid deposits within endothelial cells, smooth muscle cells of the media, and in the surrounding connective tissue, which are believed to compromise vessel wall stability and enable formation of irregular dilations of the vessels [20, 21]. Lens opacities include a spoke-like opacity of the posterior lens capsule, referred to as a “Fabry cataract”; anterior lens deposits also can occur [17].

Although conjunctival aneurysms are not described in other chronic illnesses, CV and tortuosity of retinal and conjunctival vessels are not limited to Fabry disease. Increased ocular vessel tortuosity has also been reported in patients with fucosidosis [22, 23] and GM1 gangliosidosis [24, 25]; treatment with amphiphilic drugs such as amiodarone, chloroquine, hydroxychloroquine, and indomethacin may be associated with drug-induced symmetric corneal changes that have some similarities to those seen in Fabry disease [26–28]; and lens opacities similar to Fabry cataract have been described in patients with mannosidosis and other lysosomal storage disorders [29].

The Fabry Outcome Survey (FOS) is an international registry sponsored by Shire for all patients with Fabry disease who are receiving or are candidates for ERT with agalsidase alfa. The FOS database provides information on a worldwide population of patients. A recent analysis of data from 1203 adult patients in FOS showed that patients with eye signs such as CV had more severe disease than patients without any eye signs, demonstrating a correlation between ocular manifestations of Fabry disease and disease severity [30]. The current analysis objective was to examine any correlations between eye findings and disease severity in paediatric patients in the FOS database.

## Methods

### Patients and study design

FOS is an ongoing, prospective, observational registry designed to collect clinical outcomes in patients with Fabry disease who are treatment naïve, currently treated with agalsidase alfa, or were previously treated with any ERT. For this retrospective cross-sectional analysis, data in FOS were collected from patients examined from 2001 through August 2014, with the approval of the ethical review board at each participating centre (see list in Additional file 1: Table S1). Upon enrolment into FOS, written informed consent was obtained from each patient (or parent/legal guardian for minor patients) and a physician documented each patient’s medical history, the year of diagnosis, signs and symptoms of the disease, and treatment. Depersonalised data were selected for boys (hemizygotes) and girls (heterozygotes) who underwent a detailed ocular examination, including slit-lamp examination of the anterior segment, as well as fundoscopy. The prevalence of Fabry-specific eye abnormalities was analysed in the overall FOS population, as previously described [30]. Ocular vessel tortuosity was assessed semi-quantitatively (as none, mild, moderate, or severe) by the examining eye care practitioner and was not separated between retinal and conjunctival vessels in the FOS database. FOS collects real-world data on routine clinical evaluation parameters. Therefore, no specific training was provided to practitioners to assure constancy and reliability of data collection in this multicentre study. The presence or absence of corneal haze and subepithelial corneal lines was not reported as a sign and/or symptom of Fabry disease in FOS.

### Statistical analyses

Descriptive summary statistics are presented. For categorical parameters, numbers and percentages of affected patients are reported; for continuous parameters, medians and ranges are shown. The Wilcoxon nonparametric statistic was used to compare groups. Statistical analyses were conducted using SAS V.9.2 (SAS Institute Inc., Cary, NC, USA), and data collection and analysis were supported by Shire.

Associations between eye abnormalities and severity of systemic involvement were evaluated using the disease-specific FOS Mainz severity score index (FOS-MSSI) [31, 32], which consists of four sections that cover general, neurological, cardiovascular, and renal signs and symptoms of Fabry disease [32]. Each section is weighted according to its contribution to disease morbidity, and individual organ-related scores are added to calculate the total FOS-MSSI score, which is categorised as mild (≤18), moderate (19–38), or severe (>38) [32]. Because the FOS-MSSI scoring system was not developed for and has not been validated in children, an age adjustment of the FOS-MSSI score was calculated, using an equation based on age [33, 34]. The age-adjusted FOS-MSSI disease severity scores represent the observed FOS-MSSI score minus the expected FOS-MSSI score for that patient’s age; thus, a positive age-adjusted score indicates the patient has more severe manifestations than average, and a negative age-adjusted score indicates the patient has less severe disease than average [33]. The CV subscore was removed from the total FOS-MSSI score to avoid skewing comparisons between patients with and without CV. Ocular signs were correlated with age-adjusted FOS-MSSI values.

## Results

### Baseline demographic and clinical characteristics

**Fig. 1:**
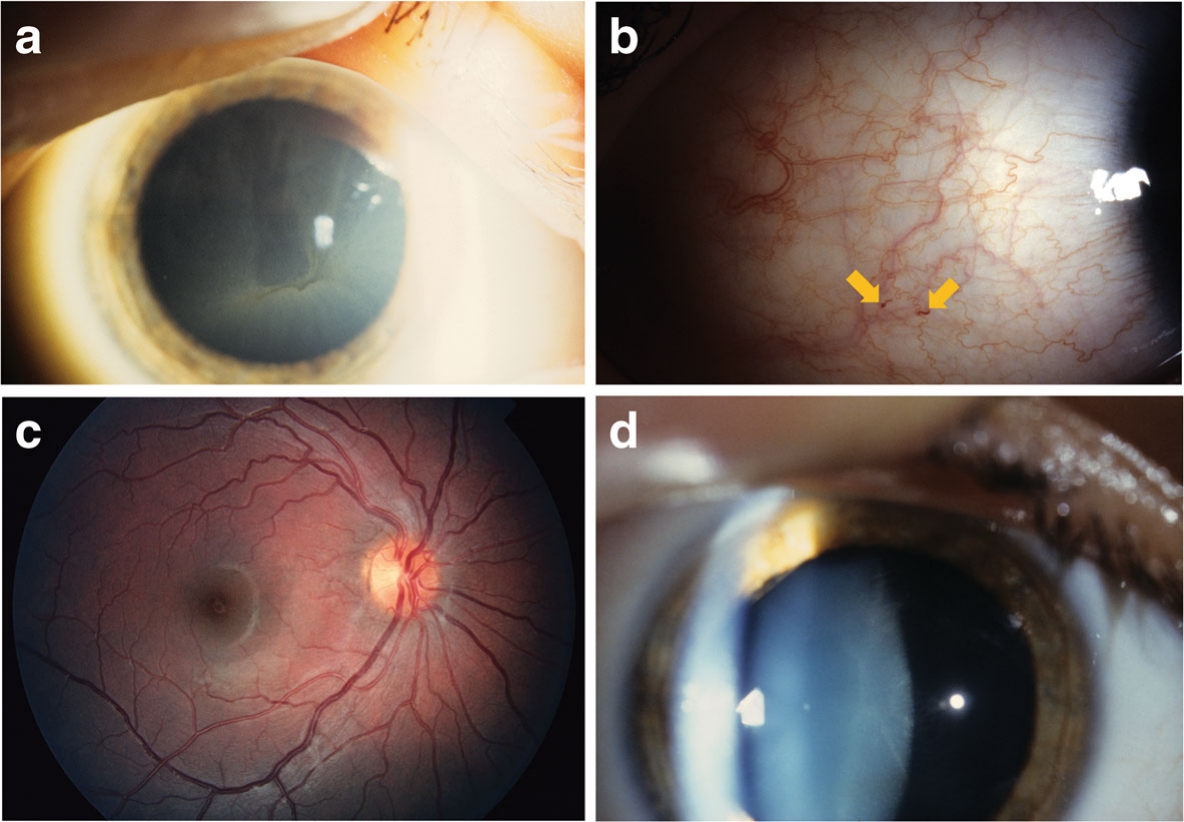
Typical ocular changes seen in Fabry disease. **a** Cornea verticillata. **b** Increased vessel tortuosity and aneurysms (arrows) in the temporal bulbar conjunctiva. **c** Increased tortuosity of arteria/vena temporalis superior. **d** Lens opacity: faint spoke-like lines at the posterior lens capsule

Among 2379 patients with data available at the August 3, 2014 extraction date, 255 were children at FOS entry; 232 underwent their first ocular examination before the age of 18 years (Table 1). At least one of the eye findings described was found in 117/232 (50.4%) children. Observed eye findings included CV (Fig. 1a) in 108/232 (46.6%), retinal and/or conjunctival vessel tortuosity (Fig. 1b, c) in 49/229 (21.4%), and posterior spoke-like lens opacity (Fig. 1d) in 5/227 (2.2%) patients. More than half (137/232; 59.1%) of the children received agalsidase alfa treatment at any time.

**Table 1:** Patient demographics and clinical characteristics

Variable	Patients		
	Boys	Girls	Total
All FOS paediatric patients with ocular exam, *n* (%)	131 (56.5)	101 (43.5)	232 (100)
Children with any eye finding, *n* (%)	62/131 (47.3)	55/101 (54.5)	117/232 (50.4)
Children with CV, *n* (%)	55/131 (42.0)	53/101 (52.5)	108/232 (46.6)
Children with tortuous vessels^a^, *n* (%)	32/131 (24.4)	17/98 (17.3)	49/229 (21.4)
Children with posterior spoke-like lens opacity, *n* (%)	2/130 (1.5)	3/97 (3.1)	5/227 (2.2)
Age of patients at first eye finding, years, median (range)	10.3 (2.3, 17.7)	8.9 (3.2, 16.4)	9.7 (2.3, 17.7)
Agalsidase alfa treatment, *n* (%)			
No	35 (26.7)	60 (59.4)	95 (40.9)
Yes (any time)	96 (73.3)	41 (40.6)	137 (59.1)
Patients with agalsidase alfa treatment and any eye finding	54 (56.3)	27 (65.9)	81 (59.1)
Patients with agalsidase alfa treatment and no eye findings	42 (43.7)	14 (34.1)	56 (40.9)

*FOS* Fabry Outcome Survey; *CV* cornea verticillata

^a^Includes both retinal and conjunctival vessel tortuosity

### Occurrence of eye findings by gender and age

Ocular signs of Fabry disease were noted in 55/101 (54.5%) girls and 62/131 (47.3%) boys who had undergone ocular examination (Table 1). CV was more common and tended to be seen at younger ages in girls than boys (seen in 52.5% of girls and 42.0% of boys; median age at first presentation, 8.9 years and 10.7 years, respectively). This is consistent with previously published reports [2, 3]. However, tortuous ocular vessels were more common and tended to be seen at younger ages in boys than girls (seen in 24.4% of boys and 17.3% of girls; median age at first finding, 10.1 years in boys and 12.0 years in girls) and reflect more severe disease; this was also seen in a previously published single-centre study [35]. Posterior spoke-like lens opacities were uncommon, being reported in only three girls and two boys.

**Fig. 2:**
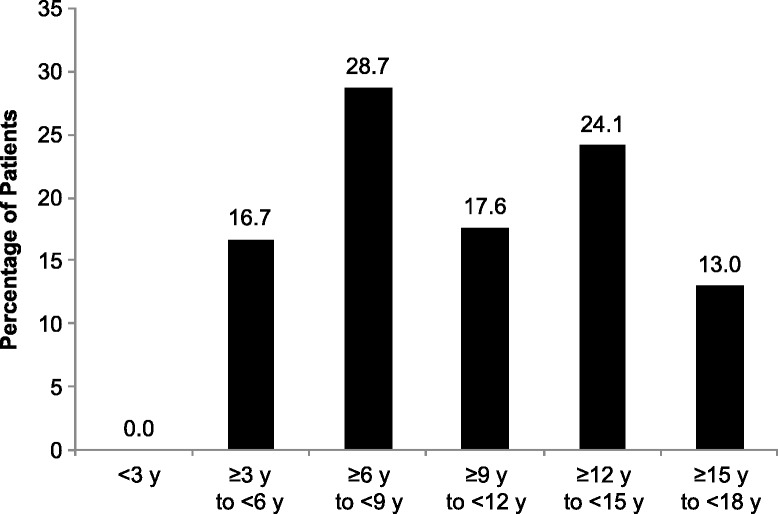
Distribution of patients by age category at first onset of cornea verticillata

Of the 232 children with ocular examinations, 108 patients had a first diagnosis of CV during the study period. The distribution of first onset of CV by age category is shown in Fig. 2.

Among the 19 children who had their last FOS-MSSI assessments before 6 years of age, four (two females) had eye findings; CV was seen in all four, tortuous vessels in two girls, and none had lens opacities. Three of the four children <6 years of age with eye findings were symptomatic with neurological signs (including hypohidrosis and/or anhidrosis). Of the 15/19 children <6 years of age with no eye findings, two were treated and six (four girls and two boys) had neurological symptoms of Fabry disease documented in FOS.

### Occurrence of eye findings by agalsidase alfa treatment status

Eighty one (59.1%) of the 137 treated paediatric patients in this study had at least one eye finding, whereas 36 (37.9%) of the 95 untreated children had any eye findings. Among the 41 treated girls, 27 (65.9%) had any eye findings versus 14 (34.1%) without eye findings (Table 1).

Of the 117 children who had eye findings, 36 (30.8%) had not received ERT during the study period, 61 (52.1%) had their first eye finding before starting therapy, and 20 (17.1%) had their first eye finding after starting therapy.

### Occurrence of eye findings by Fabry disease severity

Summary statistics for any ocular sign (including CV, tortuous retinal and/or conjunctival vessels, and posterior spoke-like lens opacity) generally showed more severe disease as measured by age-adjusted FOS-MSSI total scores in children with eye findings versus those without eye findings (median, 0.5 versus −2.3; *p* < 0.001; Table 2). These differences were statistically significant for the presence of any eye finding as well as for presence of CV and tortuous vessels (Table 2) and remained consistent when boys and girls were grouped separately. The relationship between disease severity and age at assessment by the presence (or absence) of any eye lesion is illustrated in a scatter plot (Additional file 2: Figure S1). Children with eye findings (in red) had more severe disease across all ages than children without any eye findings (in black). Disease severity increased with age in children with and without eye findings. As expected, disease severity increased with age in each group (“any eye finding” and “no eye finding”), and the differences between the two groups remained quite constant over time; the “any eye finding” group showed greater disease severity over all ages.

**Table 2:** Age-adjusted FOS-MSSI total score and eye changes

	Age-adjusted FOS-MSSI total score (removing CV), median (range)		
	With eye findings	Without eye findings	*p-*value
All patients with assessment before 18 y (*n* = 232)			
Any eye finding	0.5 (−11.0, 20.7), *n* = 117	−2.3 (−11.1, 18.8), *n* = 114	<0.001
CV	0.4 (−11.0, 20.7), *n* = 108	−2.2 (−11.1, 18.8), *n* = 123	<0.001
Tortuous vessels	3.3 (−8.7, 20.7), *n* = 49	−2.0 (−11.1, 20.5), *n* = 179	<0.001
Posterior spoke-like lens opacity	2.4 (−6.9, 20.5), *n* = 5	−1.3 (−11.1, 20.7), *n* = 221	0.257
Girls (*n* = 101)			
Any eye finding	0.9 (−9.7, 21.8), *n* = 55	−2.7 (−9.6, 9.4), *n* = 46	0.002
CV	0.9 (−9.7, 21.8), *n* = 53	−2.7 (−9.6, 9.4), *n* = 48	0.002
Tortuous vessels	2.2 (−4.6, 21.8), *n* = 17	−1.8 (−9.7, 19.3), *n* = 81	0.003
Posterior spoke-like lens opacity	1.1 (−5.9, 5.5), *n* = 3	−0.7 (−9.7, 21.8), *n* = 94	0.763
Boys (*n* = 130)			
Any eye finding	1.5 (−11.8, 19.8), *n* = 62	−2.8 (−11.9, 18.0), *n* = 68	<0.001
CV	0.3 (−11.8, 19.8), *n* = 55	−2.7 (−11.9, 18.0), *n* = 75	0.006
Tortuous vessels	3.6 (−9.4, 19.1), *n* = 32	−2.4 (−11.9, 19.8), *n* = 98	<0.001
Posterior spoke-like lens opacity	10.7 (1.7, 19.8), *n* = 2	−1.5 (−11.9, 19.1), *n* = 127	0.092

*CV* cornea verticillata, *FOS-MSSI* Fabry Outcome Survey Mainz severity score index

## Discussion

This large study on the ocular manifestations of Fabry disease confirms their presence even in some children <6 years of age and points at a prevalence of ocular involvement similar to that in the adult population. We therefore conclude that ocular examination is a valuable tool in children with Fabry disease and ocular signs may be important for further monitoring, since children with ocular changes—similar to adults with Fabry disease—show more severe disease burden. Slit-lamp examination should be routinely used whenever possible in all patients with Fabry disease.

CV is the most common ocular sign in classical Fabry disease, with a prevalence of approximately 50% among adults in FOS [30]. The reported prevalence among all ages (range, 3–71 years) in an earlier FOS study was higher, ranging from 73.1% in male patients to 76.9% in female patients [36]. Likewise, a recent systematic review of 21 published cohorts found a pooled prevalence of CV of 69% among 753 patients ranging in age from 0 to 85 years [15]. Interestingly, the prevalence of CV reported by van der Tol et al [15] was markedly lower (around 24%) when only looking at patients with non-classical Fabry disease phenotype. Allen et al reported similar findings in that patients with the cardiac variant had fewer ocular signs [35]. Previous reports indicate a similar prevalence of CV in female and male patients of all ages [18, 30], and have suggested that ocular involvement may be an indicator of disease severity. In an analysis of ocular manifestations of Fabry disease in 173 adult patients included in FOS, a correlation was observed between tortuous ocular vessels, higher FOS-MSSI values, and impaired renal and cardiac function [36]. A recent analysis of data from 1203 adult patients in FOS showed that patients with CV had more severe disease than patients without any eye signs [30]. The overall prevalence of CV in the current analysis was 46.6%, with a higher prevalence in girls (52.5%) than boys (42.0%). These findings are consistent with previous reports [6, 35].

Retinal and/or conjunctival vessel tortuosity was observed in 21.4% of this cohort. Similarly, Allen et al reported ocular vessel tortuosity in 27% of children [35]. Differences between genders have also been previously noted, with vessel tortuosity described less frequently in heterozygous female versus hemizygous male patients with Fabry disease [17, 18, 30, 36, 37]. In the present analysis, tortuous retinal or conjunctival vessels were seen in 49/229 children, which suggests that increased vessel tortuosity could be an early, specific sign of Fabry disease. Allen et al described the coexistence of symptoms of systemic autonomic neuropathy with retinal vascular changes and suggested a common pathogenesis [35]. The involvement of both the vascular and autonomic systems is likely a contributory factor for vessel alterations that can be noted even in childhood.

Future studies concerning the paediatric Fabry patient population could use the age-specific FOS Paediatric Health and Pain Questionnaire (FPHPQ), which has recently been developed and validated [38].

As expected, children who were less symptomatic were not generally receiving ERT. We observed that ocular findings have not generally been a trigger to initiate ERT, given the high percentage of children with ocular findings not on ERT. No definitive effect of ERT on eye findings has been described to date [30, 35, 36].

### Limitations of FOS

As a large international registry, FOS encompasses data from 118 clinics in 22 countries worldwide and this includes retrospective findings from a large number of eye care practitioners who likely have varying assessment strategies. FOS is a registry for patient data collected in real-world clinical settings; thus, this was not a controlled trial specifically designed to assess all parameters reported here and there were no planned scheduled assessments with all data evaluated, so only part of the information might be collected at each patient visit. Information regarding specific concurrent medications that might induce similar eye findings was not available. Furthermore, it lacks stringent inclusion and exclusion criteria seen in clinical trial settings, and thus has the potential for selection bias. In addition, the current analyses were cross-sectional in nature and, ideally, a controlled longitudinal study would be conducted to confirm these results.

## Conclusions

Routine ocular examinations are important in the care of children with Fabry disease. Because ocular signs can occur without other objective clinical signs, they remain a valuable tool in screening for Fabry disease. However, the current analysis also supports the use of ocular signs (together with other signs such as angiokeratoma and further objective clinical features such as microalbuminuria or left ventricular hypertrophy) to identify patients at risk for severe disease manifestations.

These data further emphasise, in a large cohort, the importance of ocular findings for the assessment of disease severity in children with Fabry disease. To fully understand the importance of ocular signs as predictors of disease severity, it would be necessary to evaluate further, in a systematic and prospective manner, the occurrence of ocular findings and their correlation with an efficient scoring system suited for children with Fabry disease.

## Additional files

Additional file 1: Table S1. List of ethics committees that approved the study. (PDF 30 kb)
Additional file 2: Figure S1. Scatter plot of FOS-MSSI score versus age (in years) at last FOS-MSSI score assessment, showing values for children with (in red) and without (in black) any eye findings. (PDF 964 kb)

## Abbreviations

CV: Cornea verticillata
ERT: Enzyme replacement therapy
FOS: Fabry Outcome Survey
MOS: Mainz severity score index

## References

[1] Banikazemi M, Patel M, Lemay R, Waldek S (2010). Life expectancy and cause of death in Fabry disease: findings from the Fabry Registry. Mol Genet Metab.

[2] MacDermot KD, Holmes A, Miners AH (2001). Anderson-Fabry disease: clinical manifestations and impact of disease in a cohort of 60 obligate carrier females. J Med Genet.

[3] MacDermot KD, Holmes A, Miners AH (2001). Anderson-Fabry disease: clinical manifestations and impact of disease in a cohort of 98 hemizygous males. J Med Genet.

[4] Waldek S, Patel MR, Banikazemi M, Lemay R, Lee P (2009). Life expectancy and cause of death in males and females with Fabry disease: findings from the Fabry Registry. Genet Med.

[5] Ramaswami U, Wendt S, Pintos-Morell G, Parini R, Whybra C, Leon Leal JA (2007). Enzyme replacement therapy with agalsidase alfa in children with Fabry disease. Acta Paediatr.

[6] Ramaswami U, Whybra C, Parini R, Pintos-Morell G, Mehta A, Sunder-Plassmann G (2006). Clinical manifestations of Fabry disease in children: data from the Fabry Outcome Survey. Acta Paediatr.

[7] Ries M, Gupta S, Moore DF, Sachdev V, Quirk JM, Murray GJ (2005). Pediatric Fabry disease. Pediatrics.

[8] Ries M, Ramaswami U, Parini R, Lindblad B, Whybra C, Willers I (2003). The early clinical phenotype of Fabry disease: a study on 35 European children and adolescents. Eur J Pediatr.

[9] Morier AM, Minteer J, Tyszko R, McCann R, Clarke MV, Browning MF (2010). Ocular manifestations of Fabry disease within in a single kindred. Optometry.

[10] Deegan PB, Baehner AF, Barba Romero MA, Hughes DA, Kampmann C, Beck M (2006). Natural history of Fabry disease in females in the Fabry Outcome Survey. J Med Genet.

[11] Mehta A, Ricci R, Widmer U, Dehout F, de Lorenzo Garcia A, Kampmann C (2004). Fabry disease defined: baseline clinical manifestations of 366 patients in the Fabry Outcome Survey. Eur J Clin Invest.

[12] Desnick RJ, Brady RO (2004). Fabry disease in childhood. J Pediatr.

[13] Ramaswami U, Parini R, Pintos-Morell G, Mehta A, Beck M, Sunder-Plassmann G (2006). Natural history and effects of enzyme replacement therapy in children and adolescents with Fabry disease. Fabry Disease: Perspectives from 5 Years of FOS.

[14] Samiy N (2008). Ocular features of Fabry disease: diagnosis of a treatable life-threatening disorder. Surv Ophthalmol.

[15] van der Tol L, Sminia ML, Hollak CE, Biegstraaten M. Cornea verticillata supports a diagnosis of Fabry disease in non-classical phenotypes: results from the Dutch cohort and a systematic review. Br J Ophthalmol. 2016;100(1):3–8. 10.1136/bjophthalmol-2014-306433 25677671

[16] Tsutsumi A, Uchida Y, Kanai T, Tsutsumi O, Satoh K, Sakamoto S (1984). Corneal findings in a foetus with Fabry's disease. Acta Ophthalmol (Copenh).

[17] Sher NA, Letson RD, Desnick RJ (1979). The ocular manifestations in Fabry's disease. Arch Ophthalmol.

[18] Sodi A, Ioannidis A, Pitz S, Mehta A, Beck M, Sunder-Plassmann G (2006). Ophthalmological manifestations of Fabry disease. Fabry Disease: Perspectives from 5 Years of FOS.

[19] Michaud L (2013). Vascular tortuosities of the upper eyelid: a new clinical finding in fabry patient screening. J Ophthalmol.

[20] Riegel EM, Pokorny KS, Friedman AH, Suhan J, Ritch RH, Desnick RJ (1982). Ocular pathology of Fabry's disease in a hemizygous male following renal transplantation. Surv Ophthalmol.

[21] Witschel H, Mathyl J (1969). Morphological bases of the specific ocular changes in Fabry’s disease [in German]. Klin Monatsbl Augenheilkd.

[22] Borrone C, Gatti R, Trias X, Durand P (1974). Fucosidosis: clinical, biochemical, immunologic, and genetic studies in two new cases. J Pediatr.

[23] Snodgrass MB (1976). Ocular findings in a case of fucosidosis. Br J Ophthalmol.

[24] Al-Hazzaa S, Ozand P, Traboulsi EI, Tasman W, Jaeger EA (2006). Metabolic diseases and the eye. Duane's Clinical Ophthalmology.

[25] O'Brien J (1969). Generalized gangliosidosis. J Pediatr.

[26] Falke K, Buttner A, Schittkowski M, Stachs O, Kraak R, Zhivov A (2009). The microstructure of cornea verticillata in Fabry disease and amiodarone-induced keratopathy: a confocal laser-scanning microscopy study. Graefes Arch Clin Exp Ophthalmol.

[27] Burns CA (1968). Indomethacin, reduced retinal sensitivity, and corneal deposits. Am J Ophthalmol.

[28] Yam JC, Kwok AK (2006). Ocular toxicity of hydroxychloroquine. Hong Kong Med J.

[29] Arbisser AI, Murphree AL, Garcia CA, Howell RR (1976). Ocular findings in mannosidosis. Am J Ophthalmol.

[30] Pitz C, Kalkum G, Arash L, Karabul N, Sodi A, Larroque S (2015). Ocular signs correlate well with disease severity and genotype in Fabry disease. PLoS One.

[31] Schaefer E, Mehta A, Gal A (2005). Genotype and phenotype in Fabry disease: analysis of the Fabry Outcome Survey. Acta Paediatr Suppl.

[32] Whybra C, Bahner F, Baron K, Mehta A, Beck M, Sunder-Plassmann G (2006). Measurement of disease severity and progression in Fabry disease. Fabry Disease: Perspectives from 5 Years of FOS.

[33] Hughes DA, Ramaswami U, Barba Romero MA, Deegan P (2010). Age adjusting severity scores for Anderson-Fabry disease. Mol Genet Metab.

[34] Gal A, Larroque S, Mehta A. Fabry disease: clinical severity correlates with gene mutation. Presented at: Annual Symposium of the Society for the Study of Inborn Errors of Metabolism: September 4–7 2012; Birmingham, UK; 2012.

[35] Allen LE, Cosgrave EM, Kersey JP, Ramaswami U (2010). Fabry disease in children: correlation between ocular manifestations, genotype and systemic clinical severity. Br J Ophthalmol.

[36] Sodi A, Ioannidis AS, Mehta A, Davey C, Beck M, Pitz S (2007). Ocular manifestations of Fabry's disease: data from the Fabry Outcome Survey. Br J Ophthalmol.

[37] Nguyen TT, Gin T, Nicholls K, Low M, Galanos J, Crawford A (2005). Ophthalmological manifestations of Fabry disease: a survey of patients at the Royal Melbourne Fabry Disease Treatment Centre. Clin Experiment Ophthalmol.

[38] Ramaswami U, Stull DE, Parini R, Pintos-Morell G, Whybra C, Kalkum G (2012). Measuring patient experiences in Fabry disease: validation of the Fabry-specific Pediatric Health and Pain Questionnaire (FPHPQ). Health Qual Life Outcomes.

