# Supplementary material for: Paediatric Fabry disease: prognostic significance of ocular changes for disease severity
Source: BMC Ophthalmol. 2016 Nov 16;16:202. doi: 10.1186/s12886-016-0374-2 (PMC5112699; doi:10.1186/s12886-016-0374-2)
Supplement: Additional file 1: Table S1. — List of ethics committees that approved the study. (PDF 30 kb) [file 12886_2016_374_MOESM1_ESM.pdf]

Table S1. List of ethics committees that approved the study

Ethics committee name	Country
Comisión Conjunta de Investigación en Salud – CCIS	Argentina
Comité de Ética del Instituto Médico Platense – CEDIMP	Argentina
Ethikkommission Der Medizinischen Universitat Wien und des Allgemeinen Krankenhauses der Stadt Wien AKH	Austria
Royal Melbourne Hospital HREC	Australia
Commission d'Ethique Biomedicale Hospitalo – Facultair de l'UCL	Belgium
Commission d'Ethique C.H.U. Charleroi	Belgium
Universiteit Antwerpen Ethisch Comite	Belgium
Comissão Nacional de Ética em Pesquisa (CONEP)	Brazil
Comitê de Ética em Pesquisa do Hospital de Clinicas de Porto Alegre	Brazil
Capital Health Research Ethics Board	Canada
Comité d’éthique de la recherche de l'Hôpital du Sacré-Coeur du Montréal	Canada
Comité d'éthique de la recherche en santé chez l'humain du Centre hospitalier universitaire de Sherbrooke	Canada
Conjoint Health Research Ethics Board of the University of Calgary	Canada
Health Research Ethics Authority	Canada
Health Research Ethics Board of the University of Alberta	Canada
Nova Scotia Health Authority Research Ethics Board	Canada
SickKids Research Ethics Board	Canada
The University of British Columbia	Canada
University Health Network Research Ethics Board	Canada
Vancouver Coastal Health Research Institute	Canada
Eticka komise Vseobecne fakultni nemocnice v Praze	Czech Republic
Varsinais-Suomen Sairaanhoitopiiri Eettisen toimikunnan – Hospital District of South West Finland EC	Finland
Le Comité Consultatif sur le Traitement de l'information en matière de Recherche	France
Ethikkommission an der Universitätsmedizin Greifswald	Germany
Ethikkommission der Ärztekammer Westfalen-Lippe und der Medizinischen Fakultät der WWU Münster	Germany
Ethikkommission der Landesärztekammer Baden-Württemberg	Germany
Ethikkommission der Landesärztekammer Rheinland-Pfalz	Germany
Ethikkommission der Medizinischen Fakultät der Universität Würzburg	Germany
Ethikkommission der Medizinischen Fakultät der Universität zu Köln	Germany
Egeszsegugyi Tudomanyos Tanacs Tudomanyos es Kutatasetikai Bizottsag	Hungary
Shaare Zedek Medical Center Ethics Committee	Israel
Comitato di Bioetica Fondazione I.R.C.C.S. Policlinico "San Matteo" di Pavia	Italy

Comitato Etico Area Monza Brianza	Italy
Comitato Etico Azienda Ospedaliera "Pugliese-Ciaccio" di Catanzaro	Italy
Comitato Etico Azienda Ospedaliero Universitaria, Policlinico "Paolo Giaccone" dell'Università degli Studi di Palermo	Italy
Comitato Etico Aziende Sanitarie Umbria, Perugia	Italy
Comitato Etico Azienda Unità Sanitaria Locale di Viterbo	Italy
Comitato Etico CE A.O. "Città della Salute e della Scienza" di Torino	Italy
Comitato Etico della Provincia di Ferrara	Italy
Comitato Etico Locale Azienda Ospedaliero Universitaria Careggi, Firenze	Italy
Comitato Etico per la Ricerca Biomedica Università degli Studi "G. D'Annunzio" Azienda Sanitaria Locale – Chieti	Italy
Comitato Etico per le Attività Biomediche "Carlo Romano," Università degli Studi di Napoli "Federico II"	Italy
Comitato Etico Regionale delle Marche	Italy
Comitato Etico Università Cattolica del Sacro Cuore Facoltà di Medicina e Chirurgia "Agostino Gemelli," Roma	Italy
Medisch Ethische Toetsingscommissie Universiteit van Amsterdam	The Netherlands
Comite de Etica Do Centro Hospitalar Do Alto Ave – EPE	Portugal
Komisija Republike Slovenije Za Medicinsko Etiko	Slovenia
CEIC Hospital de Torrecárdenas (FIBAO)	Spain
CEIC Complejo Asistencial de Leon	Spain
CEIC Complejo Hospitalario de Albacete	Spain
CEIC Consorcio Hospitalario Provincial de Castellón	Spain
CEIC de Andalucia (CCEIBA)	Spain
CEIC de Aragon (CEICA)	Spain
CEIC de Asturias	Spain
CEIC de Euskadi	Spain
CEIC de Galicia (CAEI)	Spain
CEIC Fundació Sant Joan de Déu	Spain
CEIC Hospital Clinic Barcelona	Spain
CEIC Hospital Clínico Universitario de Valencia	Spain
CEIC Hospital de Donostia	Spain
CEIC Hospital de Girona Dr. Josep Trueta	Spain
CEIC Hospital General de Elda	Spain
CEIC Hospital General Universitario de Alicante	Spain
CEIC Hospital General Universitario de Elche	Spain
CEIC Hospital General Universitario de Guadalajara	Spain
CEIC Hospital Universitari Arnau de Vilanova	Spain
CEIC Hospital Universitari Vall d'Hebron	Spain
CEIC Hospital Universitario Dr. Peset	Spain
CEIC Hospital Universitario Infanta Cristina	Spain
CEIC Hospital Universitari Germans Trias i Pujol	Spain
CEIC Hospital Universitario y Politecnico la Fe	Spain
CEIC Islas Baleares (CEIC-IB)	Spain
CEIC La Paz University Hospital	Spain

CEIC ASTURIAS	Spain
Fundació Puigvert	Spain
Institut d´Investigació Biomèdica de Bellvitge (IDIBELL)	Spain
Goteborgs Universitet Medicinska Fakultetens Forskningsetik Kommitte	Sweden
Commission Cantonale (VD) d'éthique de la recherche sur l'être humain	Switzerland
Kantonale Ethikkommission Zürich	Switzerland
Institutional Review Board of National Cheng Kung University Hospital	Taiwan
Institutional Review Board of Taichung Veterans General Hospital	Taiwan
Institutional Review Board of Taipei Veterans General Hospital	Taiwan
Institutional Review Board of the Veterans General Hospital Kaohsiung	Taiwan
Cambridgeshire 2 Research Ethics Committee	United Kingdom
Baylor Research Institute	United States
