# Supplementary figures and images for: Paediatric Fabry disease: prognostic significance of ocular changes for disease severity
Source: BMC Ophthalmol. 2016 Nov 16;16:202. doi: 10.1186/s12886-016-0374-2 (PMC5112699; doi:10.1186/s12886-016-0374-2)

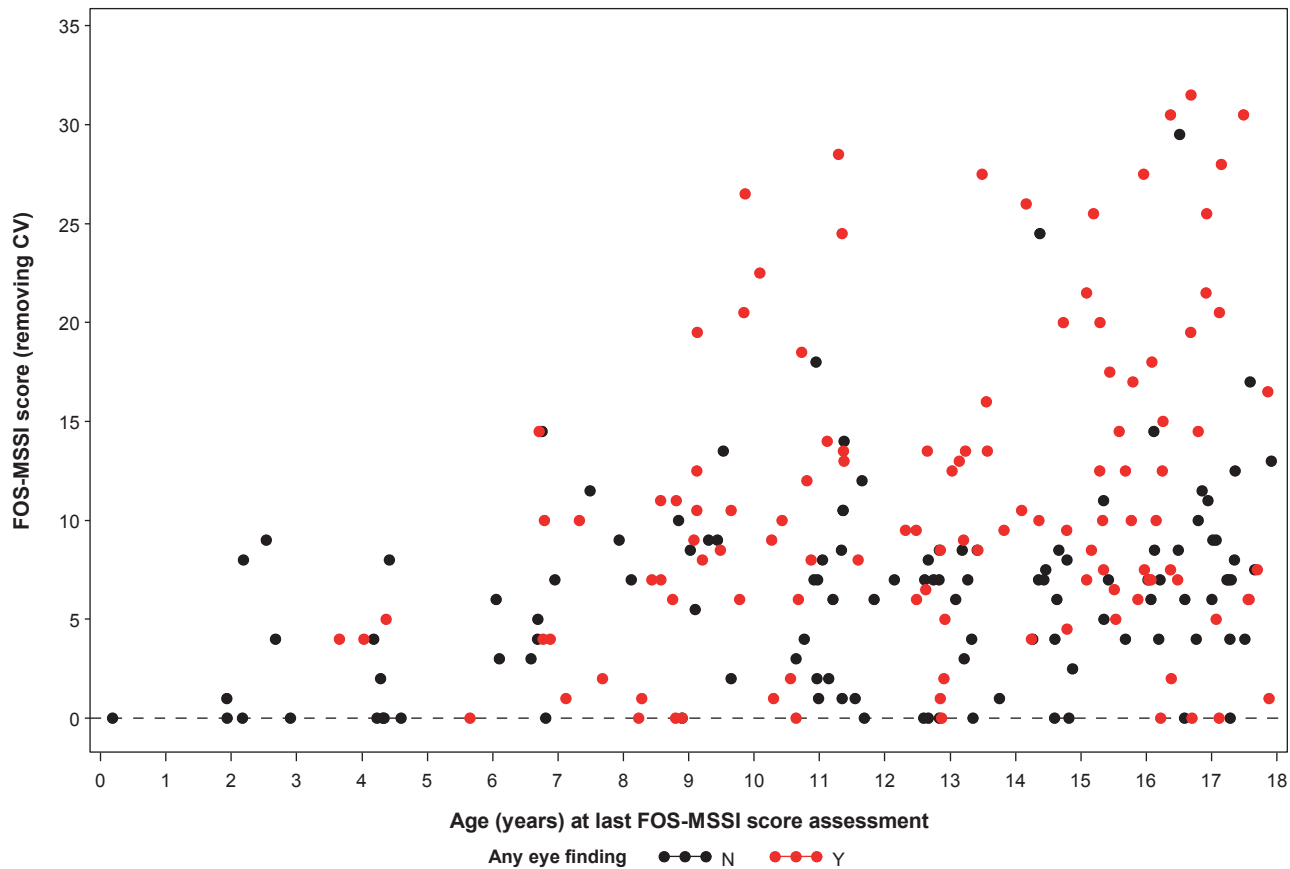

Supplement: Additional file 2: Figure S1. — Scatter plot of FOS-MSSI score versus age (in years) at last FOS-MSSI score assessment, showing values for children with (in red) and without (in black) any eye findings. (PDF 964 kb) [file 12886_2016_374_MOESM2_ESM.pdf]
